# Global analysis of H3K4me3 and H3K27me3 profiles in glioblastoma stem cells and identification of SLC17A7 as a bivalent tumor suppressor gene

**DOI:** 10.18632/oncotarget.3030

**Published:** 2015-01-22

**Authors:** Biaoyang Lin, Hwahyung Lee, Jae-Geun Yoon, Anup Madan, Elizabeth Wayner, Sanja Tonning, Parvinder Hothi, Brett Schroeder, Ilya Ulasov, Gregory Foltz, Leroy Hood, Charles Cobbs

**Affiliations:** ^1^ Cancer Institute (Key Laboratory of Cancer Prevention and Intervention, China National Ministry of Education), The Second Affiliated Hospital, Zhejiang University School of Medicine, Hangzhou, Zhejiang 310003, China; ^2^ Dept. of Urology, University of Washington, Seattle, WA 98195, USA; ^3^ System Biology Division, Zhejiang-California International Nanosystem Institute (ZCNI), Zhejiang University, Hangzhou, Zhejiang 310058, China; ^4^ Swedish Neuroscience Institute, Swedish Medical Center, Seattle, WA 98122, USA; ^5^ The Institute for Systems Biology, Seattle, WA 98109, USA; ^6^ LabCorp Clinical Trials (Genomics Laboratory), Seattle, WA 98109, USA

**Keywords:** H3K4me3, H3K27me3, glioblastoma, stem cells, SLC17A7

## Abstract

Epigenetic changes, including H3K4me3 and H3K27me3 histone modification, play an important role in carcinogenesis. However, no genome-wide histone modification map has been generated for gliomas. Here, we report a genome-wide map of H3K4me3 and H3K27me3 histone modifications for 8 glioma stem cell (GSC) lines, together with the associated gene activation or repression patterns. In addition, we compared the genome-wide histone modification maps of GSC lines to those of astrocytes to identify unique gene activation or repression profiles in GSCs and astrocytes. We also identified a set of bivalent genes, which are genes that are associated with both H3K4me3 and H3K27me3 marks and are poised for action in embryonic stem cells. These bivalent genes are potential targets for inducing differentiation in glioblastoma (GBM) as a therapeutic approach. Finally, we identified SLC17A7 as a bivalent tumor suppressor gene in GBM, as it is down-regulated at both the protein and RNA levels in GBM tissues compared with normal brain tissues, and it inhibits GBM cell proliferation, migration and invasion.

## INTRODUCTION

Epigenetic changes, including histone modification, play an important role in carcinogenesis. There are several types of histone marks, including histone methylation and acetylation. In this manuscript, we focus on two prominent histone methylation marks: H3K4me3 (trimethylated lysine 4 on histone H3) and H3K27me3 (trimethylated lysine 27 on histone H3). H3K4me3 is associated with active transcription and is catalyzed by Trithorax protein complexes, whereas H3K27me3 is associated with silenced genes and is catalyzed and maintained by Polycomb Repressive Complex 2 (PRC2) [1, 2].

With the recent development of ChIP-chip (chromatin immunoprecipitation coupled with DNA chip analysis) and ChIP-seq (chromatin immunoprecipitation coupled with next-generation sequencing) technologies, genome-wide epigenetic marks have been studied extensively in the embryonic stem cells of humans and mice [1–5]. Our group and others have applied ChIP-chip and ChIP-seq to analyze genome-wide epigenetic marks in cancer cells, such as those of prostate cancer [6, 7]. However, no genome-wide histone modification map has been generated for gliomas. Here, we applied ChIP-seq to create a genome-wide map of histone modifications in 8 glioma stem cell (GSC) lines that we generated in the laboratory and associated these modifications with gene activation or repression patterns. Furthermore, we compared the genome-wide map of histone modifications in GSC lines with those in astrocytes to identify unique gene activation or repression profiles in GSC lines.

Bivalent genes, which are characterized by the presence of both H3K4me3 and H3K27me3 modifications, have been heralded as a hallmark of ESCs [1, 8]. Bivalent genes were initially identified in a subset of key developmentally regulated genes in embryonic stem cells (ESCs) and are thought to prime genes for activation while keeping them repressed [9]. We hypothesized that cancer stem cells (CSCs), in our case, glioma stem cells (GSCs), utilize comparable bivalent binding regions as an epigenetic control mechanism similar to that observed in ESCs. Identifying and understanding these bivalent binding regions in GSCs would help us to understand the properties of cancer stem cells, including how GSCs escape anti-proliferative drugs or cell differentiation to maintain their stem cell-like properties, and to design epigenetic therapeutic approaches to overcome cancer stem cells in cancers. In this study, we identified a set of bivalent genes for GSCs. These bivalent genes are potential targets for inducing differentiation in GBM as a therapeutic approach. Finally, we identified SLC17A7 as a bivalent tumor suppressor gene in GBM by demonstrating its down-regulation at both the protein and RNA levels in GBM tissues compared with normal brain tissues.

## RESULTS

### Global analysis of H3k4me3 and H3k27me3 profiles in glioma stem cells (GSCS) and astrocytes

We established glioma stem cell (GSC) lines from GBM tissues using the method developed by Pollard et al. [10]. In brief, tumor samples were treated with Accutase (Sigma, St. Louis, MO) immediately after surgical resection, and the single cell suspension was plated in NeuroCult® NS-A medium with epidermal growth factor (EGF) and fibroblast growth factor 2 (FGF-2). These GSCs contained cells that retained cancer-initiating stem cell properties. Two of the GSC lines (SN186 and SN184) formed tumors when 1 million cells were injected into mice in a subcutaneous model [11]. Therefore, we used SN186 data as representative data to illustrate our observations for the H3K4me3 and H3K27me3 profiles in this study.

**Figure 1:**
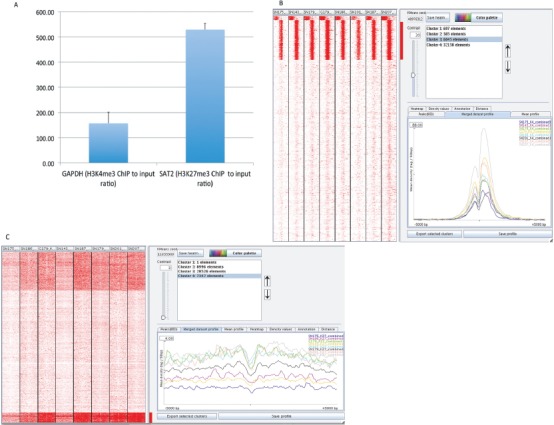
Global identification of H3K4me3 and H3K27me3 binding profiles in glioma stem cells (GSCs) **(A)** Quantitative ChIP-PCR of the representative H3K4me3-marked gene GAPDH and the representative H3K27me3-marked gene SAT2. GAPDH, a house-keeping gene and positive control for H3K4me3 ChIP, was found to be enriched 118-fold, whereas SAT2 (satellite 2), a gene present in heterochromatin and a positive control for H3K27 methylation, was found to be enriched 528-fold (Y-axis, fold enrichment for the H3K4me3 or H3K27me3 ChIP DNAs versus the input DNA). **(B)** The peak density heat map for H3K4me3 around TSSs (transcription start sites). The whole set of TSSs from 39,865 RefSeq genes from UCSC is shown for the 8 GSC lines after clustering into 5 groups using the SeqMINER program. TSSs were used as reference coordinates. Tag densities within a window of +/−5 kb on either side of the TSS coordinates were collected from each ChIP-seq dataset. The right panel shows the H3K4me3 peak profiles in the 8 GSC lines for the genes in cluster 2, which is one of the clusters enriched for H2K4me3. The H3K4me3 profile exhibits a strong dual peak with a central notch. The plot was generated using the SeqMINER program. **(C)** The peak density heat map of H3K27me3 around the complete set of TSSs (transcription start sites) from 39,865 RefSeq genes from UCSC for the 8 GSC lines. Tag densities within a window of +/− 5 kb on either side of the TSS coordinates were collected from each ChIP-seq dataset. The peaks are much broader than those for H3K4me3.

We performed ChIP with antibodies against H3K4me3, H3K27me3 and H3 in eight GSC lines (SN143, SN175, SN179, SN186, SN187, SN201, G179 and SN207) and, for comparison, an astrocyte cell line obtained from ScienCell Inc. (Cat# 1800, Carlsbad, CA). DNA isolated by chromatin IP with anti-H3K4me3, anti-H3K27me3 or anti-H3 antibodies was subjected to next-generation sequencing (ABI5500, Life Technologies, Carlsbad, CA). In addition, total genomic DNA (‘input DNA’) from each sample was sequenced. To assess the quality of the ChIP procedure, we performed quantitative PCR to assess the enrichment of known target genes. GAPDH, a house-keeping gene and positive control for H3K4me3 ChIP [12], was found to be enriched more than 100-fold. For example, GAPDH was enriched 157-fold in the H3K4me3 ChIP from the SN186 GSC cell line versus the IgG control ChIP (Figure 1A). The satellite 2 (SAT2) gene, which is present in heterochromatin and is a positive control for H3K27 methylation, was found to be enriched several hundred-fold over the IgG control ChIP (e.g., 529-fold in the SN186 GSC line, Figure 1A). These data demonstrated the efficacy of our histone modification ChIP protocol. The ChIP-processed DNA and input DNA were then subjected to next-generation sequencing as previously described [13].

The raw sequence reads for each cell line were aligned to the human genome (hg19) using the Bowtie alignment program (bowtie-bio.sourceforge.net/). The number of aligned reads for each ChIP condition (H3K4me3, H3K27me3, and H3) and the input DNA are tabulated in Supplementary Table 1. To assess the general quality of the data generated, we used the seqMINER program [14] to visualize and cluster the raw sequence reads 5 kb upstream and 5 kb downstream of the transcription start sites (TSS) of 39,865 RefSeq genes. Figure 1B shows the read densities clustered into 4 groups at regions around the TSS (+/−5 kb) for the H3K4me3 profiles of the eight GSC lines. The genes in clusters 1–3 are those with enriched H3K4me3 peaks. The read densities at these genes were highest around their transcription start sites, which is consistent with the association between H3K4me3 and active promoters. Their profiles exhibit a strong dual peak with a central notch, similar to the typical profile of H3K4me3 [1–5], as illustrated in Figure 1B (lower right panel).

By contrast, the H3K27me3 profiles exhibit peaks around the TSS sites that are much broader than those observed in the H3K4me3 profiles. Figure 1C shows the read densities at regions around the TSS (+/−5 kb) for the H3K27me3 profiles of the eight GSC lines clustered into 4 groups. Again, the profiles obtained for H3K4me27 in GSC lines are similar to those previously observed in other cell types [1–5, 12].

### Identification of H3K4me3- and H3K27me3-associated genes in glioma stem cells (GSCs) and astrocytes

To utilize the sequencing data from input DNA (controls) that we generated for each sample, we decided to use SICER (a clustering approach for identification of enriched domains from histone modification ChIP-Seq data) [15], to analyze each sample individually for H3K4me3 and H3K27me3 patterns and to incorporate the appropriate control data. Significant peaks were called with an FDR of less than 1E-3.

**Figure 2:**
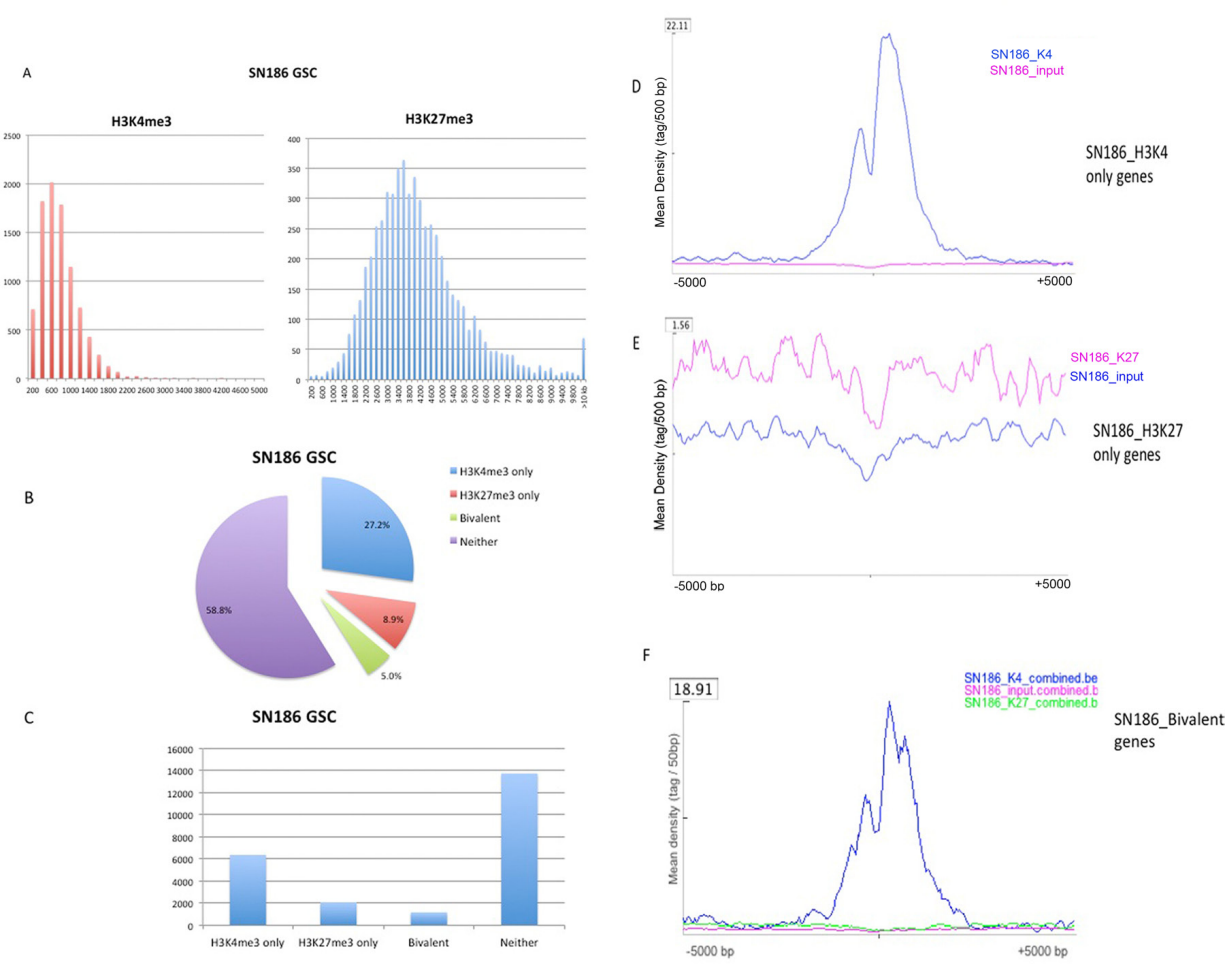
Properties of the H3K4 and H3K27 trimethylation patterns illustrated by the SN186 GSC line **(A)** The frequency distribution of peaks in bins of 200 bp is shown for H3K27me3 and H3K27me3 (X-axis, bins of 200 bp; Y-axis, the number of peaks in each bin). **(B)** The percentage of genes in the H3K4me3, H3K27me3, bivalent, and unmodified categories in GSC line SN186. **(C)** Number of genes identified in the H3K4me3, H3K27me3, bivalent, and unmodified categories in GSC line SN186. **(D)** The peak density profiles around TSSs (+/− 5kb) for H3K4me3-only genes in GSC line SN186. **(E)** The peak density profiles around TSSs (+/− 5kb) for H3K27me3-only genes in GSC line SN186. F, The peak density profiles around TSSs (+/− 5kb) for bivalent genes in GSC line SN186.

Data from the SN186 GSC line, a cell line that fhas been shown to form tumors in mice [11], illustrate the features of the H3K4 and H3K27 trimethylation patterns in GSC lines. For SN186, we identified 9183 H3K4me3 peaks (corresponding to 8099 RefSeq genes and 11 unannotated peaks) and 5965 H3K27me3 peaks (corresponding to 3569 RefSeq genes and 4 unannotated peaks). Of these peaks, 7488 H3K4me peaks (81.5%, 7488/9183) have lengths of less than 1 kb, with a median at 799 bp. The frequency distributions of peaks in bins of 200 bp are shown in Figure 2A (left panel). For H3K27me3, the peak lengths are much broader (median 3799) than those for H3K4me3, as shown by the frequency distribution (Figure 2A, right panel). For H3K27me3 peaks, the most frequent peak lengths were between 3 kb and 4 kb (approximately 33%), and most peaks (63%) were in the size range of 2.6–5 kb (Figure 2A, right panel). Of the 23393 unique known RefSeq genes (from UCSC hg19), 6374 (27.2%) have H3K4me3 peaks only, while 2090 (8.9%) genes have H3K27me3 peaks only, and 1175 (approximately 5%) are bivalent (having both H3k4me3 and H3K27me3 peaks) (Figure 2B–2C).

We plotted the peak density profiles around the TSSs for H3K4me3-only genes, H3K27me3-only genes, bivalent genes, and genes in none of these categories for the H3K4me3 and H3K27me3 ChIP-seq profiles and the input DNA sequencing data from the SN186 cell line. We found that the H3K4me3 genes are distinguished by sharp peaks, with a mean peak density of 22.11 tags per 50 bp (Figure 2D), whereas the H3K27me3 peaks are much broader, with a mean density of 1.56 tag per 50 bp (Figure 2E). The bivalent peak genes have a mean peak density of 18.91 tags per 50 bp (Figure 2F).

**Figure 3:**
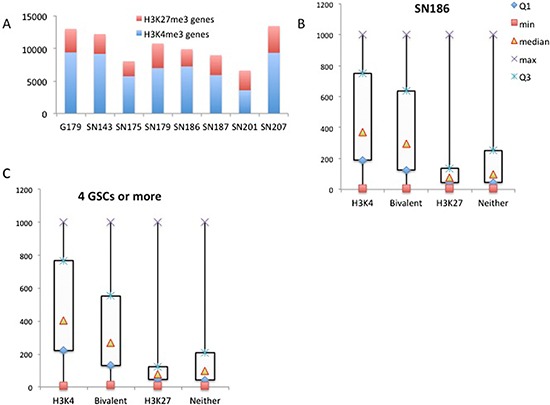
Correlation of H3K4 and H3K27 trimethylation with gene activity **(A)** The number of genes with H3K4me3 or H3K27me3 marks for the 8 GSC lines (X-axis, the number of genes). **(B)** A box plot of gene expression levels for the genes in the H3K4m3-only, H3K27me3-only, bivalent and unmodified categories in SN186 cells (Y-axis, normalized intensities from the array hybridization). **(C)** A box plot of gene expression levels for the genes in the H3K4m3-only, H3K27me3-only, bivalent and unmodified categories in more than four of the eight glioma stem cell lines (Y-axis, normalized intensities from array hybridization).

We analyzed the data from the remaining 7 GSC lines with the SICER program using the same criteria as we described for SN186. Figure 3A shows the numbers of genes with H3K4me3 or H3K27me3 marks for the 8 GSC lines. We noticed that there were heterogeneities between the GSC lines with regard to the number of H3K4me3- and H3K27me3-marked genes. By combining the data from all 8 GSC lines, we identified 9808 genes that contained H3K4me3 peaks in at least 4 of the 8 GSC lines (Supplementary Table 2) and 4612 genes that contained H3K27me3 peaks in at least 4 of the 8 GSC lines (Supplementary Table 3).

We were interested in comparing the H3K4me3 and H3K27me3 profiles in GSC to those in the human embryonic stem cells (hESC). There are several H3K4me3 and H3K27me3 data set published for human embryonic stem cells [3, 4, 16, 17]. As Guenther et al. conducted the analysis for six hESCs, it is the most comprehensive analysis of the H3K4me3 and H3K27me3 profiles for hESC. We therefore compared our data for GSC with their data. For H3K4me3, there are 13,239 genes that have H3K4me3 peaks in at least 3 of 6 hESCs [16]. 8,365 of these genes (63.2%) were also marked by H3K3me3 in more than 4 of 8 GSCs (Supplementary Table 4, sheet 1). For H3K27me3, there are 2,425 genes that have H3K4me3 peaks in at least 2 of 5 hESCs (only 5 hESCs have the H3K27me3 profile data) [16]. 1,151 (47.5%) of them were also bound by H3K27me3 in more than 4 of 8 GSCs (Supplementary Table 4, sheet 2). These observations suggested a significant difference in the H3K4me3 and H3K27me3 profiles between GSC and hESCs. This is in contrast with the observation that little difference in these profiles was found between ESCs and iPSCs [16].

We were particularly interested in those genes that are uniquely marked in GSCs comparing to hESCs. We found that there are 781 genes (Supplementary Table 4, sheet 1) that are uniquely marked by H3K4me3 in GSCs and 2835 genes (Supplementary Table 4, sheet 2) that are uniquely marked by H3K27me3 in GSCs comparing with hESCs. Functional annotations of these genes revealed that the GO term GO:0035556 intracellular signal transduction is marked by active H3K3me3 modification (data not shown). The GO term GO:0006955 immune response and GO:0006952 defense response were marked by the repressive H3K27me3 modifications (data not shown).

### Correlation of H3K4 and H3K27 trimethylation with gene activity

Because H3K4me3 is associated with active promoters and H3K27me3 is associated with silenced promoters, we were interested in the relationship between gene expression activity and trimethylation of H3K3 and H3K27. We compared expression array data on the 8 GSC lines with the H3K4 and H3K27 trimethylation patterns. Using SN186 as an example, we found that H3K4me3-marked genes have the highest median expression levels, while those marked by H3K27me3 have the lowest median expression levels (Figure 3B). The bivalent genes (marked by both H3K4me3 and H3K27me3) have intermediate expression levels (Figure 3B). We also compared the H3K4 and H3K27 trimethylation patterns of genes that are found in 4 or more of the 8 GSC lines and found that the correlation with gene expression was similar to that found in the SN186 line (Figure 3C). These findings are consistent with previously observed correlations and patterns of gene expression for H3K4me3- and H3K27me3-marked genes [3].

### Differences in the genes marked by H3K4 and H3K27 trimethylation between GSCs and astrocytes

**Figure 4:**
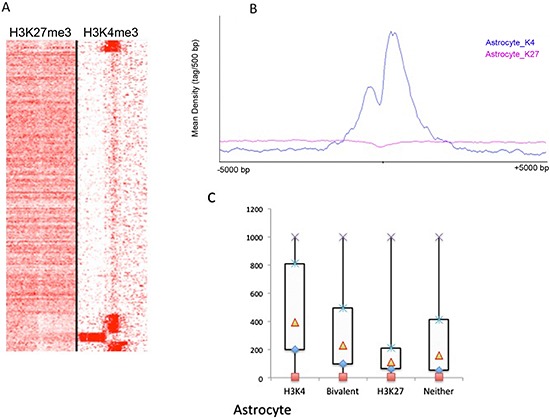
Global identification of H3K4me3 and H3K27me3 binding profiles in astrocytes **(A)** The peak density heat map for H3K4me3 and H3K27me3 around the TSSs (transcription start sites) of the complete set of 39,865 RefSeq genes from UCSC. Tag densities within a window of +/− 5 kb on either side of the TSS coordinates were collected from each ChIP-seq dataset. **(B)** The peak density profiles around TSSs (+/− 5kb) for H3K27me3 and H3K27me3 in astrocytes. **(C)** A box plot of gene expression levels for the genes in the H3K4m3-only, H3K27me3-only, bivalent and unmodified categories in astrocytes (Y-axis, normalized intensities from array hybridization).

We also analyzed astrocytes and compared them with GSCs. For astrocytes, characteristic H3K4me3 and H3K27me3 peak profiles were also observed (Figure 4A, 4B). After SICER analysis, we identified 4483 H3K4me3 peaks (corresponding to 3941 RefSeq genes and 12 unannotated peaks, Supplementary Table 5) and 9742 H3K27me3 peaks (corresponding with 4548 RefSeq genes and 4 unannotated peaks, Supplementary Table 6). We identified approximately twice as many H3K27me3 genes (repressed) as H3K4me3 genes (activated), suggesting that there are more genes being repressed rather than activated in astrocytes. This is in contrast with GSC lines, for which we identified approximately twice as many H3K4me3 genes as H3K27me3 genes (Figure 3A). This observation is in line with expectations when considering that GSCs are stem cell-like cells and thought to have greater numbers of actively expressed genes than observed in astrocytes, which are differentiated cells and might express only genes with specialized functions. Again, in astroctes, the patterns of expression levels for genes in different categories of trimethylation were similar to that found in the SN186 line (Figure 4C).

We were interested in genes marked by H3K4 and H3K27 trimethylation in GSCs (in more than 4 of the 8 GSC lines) but not in astrocytes. We identified 6593 H3K4me3-marked genes (Supplementary Table 5) and 1939 H3K27me3-marked genes (Supplementary Table 6) that are uniquely marked in GSC versus astrocytes. Conversely, we also analyzed the data to identify those genes that are marked by trimethylation in astrocytes but not in GSC lines. We identified 773 H3K4me3-marked genes (Supplementary Table 5) and 2662 H3K27me3-marked genes (Supplementary Table 6) that are uniquely marked in astrocytes versus GSC lines.

### Identification of bivalent genes that are differentially expressed between GSCs and astrocytes

In GSCs, bivalent genes, which are defined by the presence of both H3K4me3 and H3K27me3 marks, could be subject to epigenetic control and amenable to therapeutic manipulation. We therefore focused on the identification of those bivalent binding regions defined by H3K4me3 and H3K27me3 peaks that are within 10 kb of each other. We identified a total of 317 genes that have bivalent peaks within 10 kb of each other in at least 4 of the 8 GSC lines (Supplementary Table 7), and these included many previously identified bivalent genes. For example, SOX11, a previously identified bivalent gene [18], was also identified as a bivalent gene in GSC lines.

Several genes associated with the WNT pathway were found to be bivalent. These included TCF3 (transcription factor 3, E2A immunoglobulin enhancer binding factors E12/E47), WNT2 (wingless-type MMTV integration site family member 2), WNT16 (wingless-type MMTV integration site family member 16), and BMP6 (bone morphogenetic protein 6), which suggests that the WNT pathways are regulated by bivalent modification in GSCs.

Other interesting classes of bivalent genes include the HOX gene family, represented by the genes HLX (H2.0-like homeobox), HOXA3, HOXB6, HOXB9, HOXC11 and 12 additional homeobox containing genes (Table 1), and the forkhead box gene family, represented by FOXA3, FOXJ2, and FOXK1 (Table 1). In addition, nine genes involved in potassium channel function and 5 genes involved in solute carrier function were also found to be bivalent.

**Table 1:** Interesting genes that are bivalent in more than 4 out 8 GSCs

Gene Names	Numbers of GSCs that are bivalent	Descriptions
**WNT pathway genes**		
WNT16	5	Wingless-type MMTV integration site family, member 16
WNT2	4	Wingless-type MMTV integration site family member 2
TCF3	6	Transcription factor 3 (E2A immunoglobulin enhancer binding factors E12/E47)
BMP6	4	Bone morphogenetic protein 6
**Potassium channel genes**		
KCNA4	6	Potassium voltage-gated channel, shaker-related subfamily, member 4
KCNC3	4	Potassium voltage-gated channel, Shaw-related subfamily, member 3
KCNG2	4	Potassium voltage-gated channel, subfamily G, member 2
KCNK6	4	Potassium channel, subfamily K, member 6
KCNN1	5	Potassium intermediate/small conductance calcium-activated channel, subfamily N, member 1
KCNN3	4	Potassium intermediate/small conductance calcium-activated channel, subfamily N, member 3
KCP	4	
KCTD15	4	Potassium channel tetramerisation domain containing 15
**Solute carrier family genes**		
SLC17A7	4	Solute carrier family 17 (sodium-dependent inorganic phosphate cotransporter), member 7
SLC19A1	5	Solute carrier family 19 (folate transporter), member 1
SLC35C1	4	Solute carrier family 35, member C1
SLC5A5	5	Solute carrier family 5 (sodium iodide symporter), member 5
SLC8A2	5	Solute carrier family 8 (sodium-calcium exchanger), member 2
**Homeobox family genes**		
HLX	4	H2.0-like homeobox
HOXA3	4	Homeobox A3
HOXB6	4	Homeobox B6
HOXB9	4	Homeobox B9
HOXC11	4	Homeobox C11
LHX1	7	LIM homeobox 1
LHX4	5	LIM homeobox 4
LHX9	4	LIM homeobox 9
MEIS1	5	Meis homeobox 1
MNX1	5	Motor neuron and pancreas homeobox 1
NOTO	4	Notochord homeobox
SIX3	4	SIX homeobox 3
TLX2	5	T-cell leukemia homeobox 2
UNCX	5	UNC homeobox
CUX1	7	Cut-like homeobox 1
EN2	6	Engrailed homeobox 2
EVX2	4	Even-skipped homeobox 2

Gene ontology analysis of the GSC bivalent gene set revealed that it is enriched for genes involved in developmental processes (tissue development and organ development) and cell differentiation (data not shown), which is consistent with the roles of the bivalent genes identified in embryonic stem cells.

We compared the bivalent genes of GSC lines with those of astrocyte cells (393 bivalent genes, Supplementary Table 8) and found 255 bivalent genes that were unique to our GSC lines, while only 62 bivalent genes were found to be common to both GSCs and astrocytes. This suggests that the bivalent program in GSCs is different from that in astrocytes. The uniquely bivalent genes in GSCs could be explored as potential therapeutic targets in gliomas.

### Bivalency of putative tumor suppressor genes and the identification of SLC17A7 as a bivalent tumor suppressor gene

We were interested in identifying genes that are bivalent in GSCs and that have a higher expression in GSCs than in astrocytes, with the hypothesis that knocking down the expression of these bivalent genes in GSCs would induce the differentiation of GSCs toward the astrocyte lineage. Such a mechanism could be used as a therapeutic approach for GBM. To identify these genes, we integrated the expression array data obtained for bivalent genes in GSCs and astrocytes.

**Figure 5:**
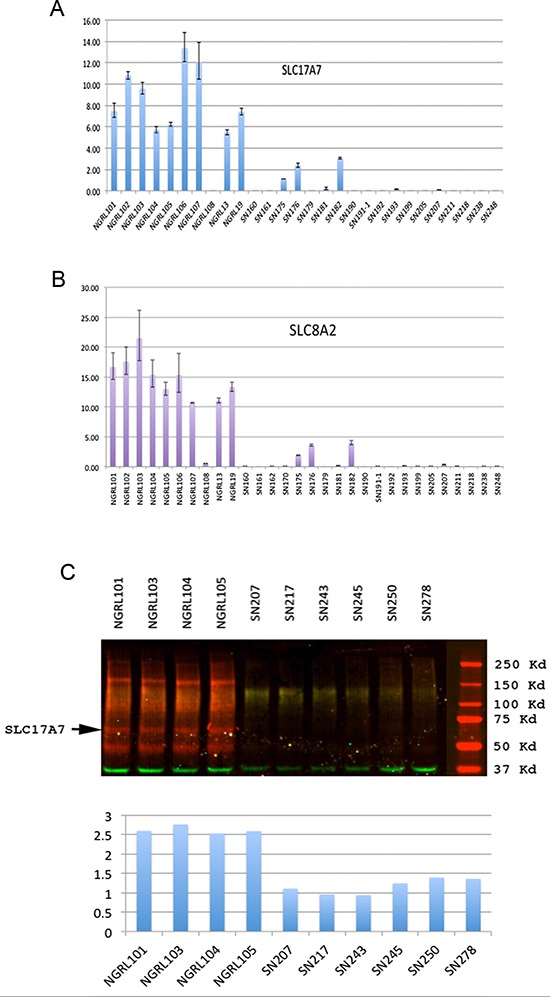
Analysis of bivalent tumor suppressors in GBM **(A)** and **(B)**, Real-time PCR expression analysis of two putative bivalent tumor suppressors SLC17A7 **(A)** and SLC8A2 **(B)** in GBM tissues and normal brain tissues (Y-axis, relative expression levels from quantitative RT-PCR; NGRL series, normal brain tissues; SN series, GBM tissues). **(C)** Western blot analysis of SLC17A7 expression in GBM tissues and normal brain tissues. Two-color Western blot analysis using the LI-COR Odyssey IR imaging system. The red channel shows the signal from the SLC17A7 antibody, and the green channel shows the signal from the antibody for the control protein GAPDH. The bottom is the quantification of the Western blot results.

It has been proposed that many tumor suppressors are preferentially pre-marked in ESCs as bivalent genes poised for silencing in human cancers (e.g., via CpG island hypermethylation) [19]. We have focused on the identification of bivalent tumor suppressor in this manuscript. To search for candidate bivalent tumor suppressor genes, we looked for bivalent genes that are down-regulated in GBM tissue compared with normal brain tissue. For this purpose, we analyzed the expression levels of our bivalent gene set in the TCGA GBM and normal brain tissue array data (www.TCGA.org). We identified SLC17A7 and SLC8A2 as two of the top nine genes that were down-regulated by more than 1.8-fold in GBM tissue compared with normal brain tissue. Using RT-PCR, we confirmed the down-regulation of four putative bivalent tumor suppressor genes, SLC17A7 [Solute carrier family 17, member 7] and SLC8A2 [Solute carrier family 8, member 2] (Figure 5A–5B), in an independent panel of GBM tissues compared with a panel of normal brain tissues in our laboratory.

An analysis of the epigenetic regulation of SLC8A2 in glioma has been performed previously [20]. We therefore focused on the analysis of SLC17A7. We conducted a Western blot analysis and found that SL17A7 protein was also under-expressed in GBM tissues compared with normal brain tissues (Figure 5C).

### SLC17A7 overexpression reduces proliferation, migration and invasion of GBM cells

**Figure 6:**
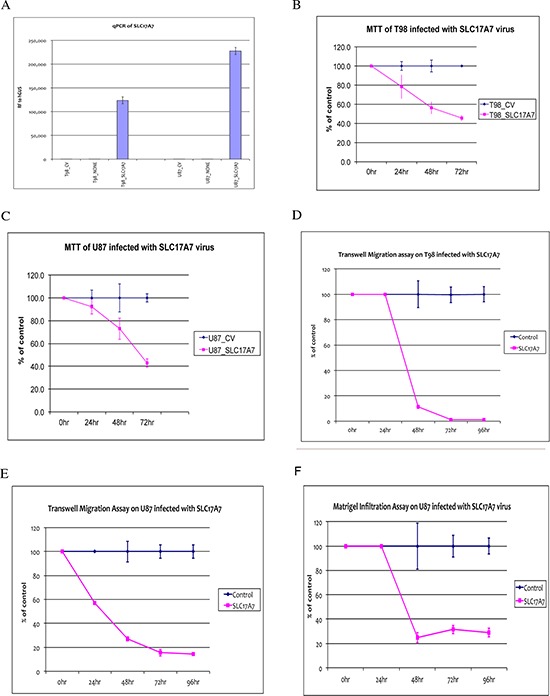
Functional Analysis of SLC17A7 in GBM cells **(A)** RT-PCR confirmation of overexpression of SLC17A7 in T98 and U87 infected with SLC17A7 adenovirus (T98_SLC17A7 or U87_SLC17A7) compared with their MOCK-controls (T98-CV or U87-CV) or non-infected parental cells (T98-NONE or U87-NONE). **(B)** and **(C)** MTT assays comparing cell proliferation rates between T98 and U87 infected with SLC17A7 adenovirus (T98_SLC17A7 or U87_SLC17A7) and their MOCK-controls (T98-CV or U87-CV) for a time course till 72 hours after infection. **(D)** and **(E)** Transwell migration assays comparing cell migration capabilities between T98 and U87 infected with SLC17A7 adenovirus (T98_SLC17A7 or U87_SLC17A7) and their MOCK-controls (T98-CV or U87-CV) for a time course till 96 hours after infection. **(F)** Matrigel invasion assay comparing U87 infected with SLC17A7 adenovirus (U87_SLC17A7) and its MOCK-controls (U87-CV) for a time course till 72 hours after infection.

We generated SLC17A7 overexpression construct via Vector BioLabs (Philadelphia, PA) using Adenoviral - Type 5 (dE1/E3) backbone vector and the CMV promoter. Adenoviral packaging and amplification were both performed by Vector BioLabs. Figure 6A showed that SLC17A7 is overexpressed after transfecting the U87 and T98 cells with the adenovirus constructs. MTT assay showed that SLC17A7 overexpression slowed down cell proliferation rate to about 40% that of the control (MOCK transfection) (Figure 6B–6C). Transwell migration and *in vitro* Matrigel infiltration assay showed that SLC17A7 overexpression dramatically reduced the cell migration (Figure 6D–6E) and invasion capabilities (Figure 6F).

## DISCUSSION

We conducted a comprehensive analysis of the global patterns of two key histone methylation marks, H3K4me3 and H3K27me3, in glioma stem cells and astrocytes. To our knowledge, this is the first comprehensive analysis of histone methylation in glioma stem cells. However, because the original aim of our study was to identify bivalent genes containing both H3K4me3 and H3K27me3 (as key regulators of glioma carcinogenesis), the study does not include an analysis of the patterns of other types of histone methylation, such as H3K9 or H3K36 or the mono- or di-methylation patterns of H3K4 and H3K27.

Because many tumor suppressors are preferentially pre-marked in ESCs as bivalent genes poised for silencing in human cancers (e.g., via CpG island hypermethylation) [19], we focused on the analysis of bivalent genes that are putative tumor suppressors. Solute carrier family members have previously been identified as tumor suppressors. For example, SLC5A8 was identified as a tumor suppressor gene that is frequently down-regulated by promoter hypermethylation in prostate tumors [21] and pancreatic cancer [22]. SLC19A3 is down-regulated via promoter methylation in gastric cancer [23]. Lindqvist et al. found that methylation in the CpG islands of the SLC25A43 gene could be an alternative mechanism of gene silencing in the absence of LOH in breast cancer [24]. We identified SLC8A2 as a bivalent gene that is down-regulated in GBM compared with normal brain tissues. SLC8A2 is encoded on chromosome 19q13.3, and loss of heterozygosity at 19q13.3 is both a common genetic change in human gliomas [25] and a prognostic marker for predicting overall survival [26]. Previously, many tumor suppressor genes associated with gliomas have been found to localize to this region, including p190-A [27], a Ras GAP-binding phosphoprotein of 190 kDa. Many genes localized to this region are epigenetically silenced, including paternally expressed imprinted gene 3 (PEG3) [28], epithelial membrane protein 3 (EMP3) [29], and HSS1 (hematopoietic signal peptide-containing secreted 1) [30]. Qu et al. recently performed an epigenetic analysis of SLC8A2 in glioma [20]. They found that although the CpG island in the 5′ promoter region of SLC8A2 was unmethylated, the 5′ CpG-rich area (the so-called CpG island shore) was methylated, as were the CpG sites of three gene-body CpG islands located in exon 2, introns 2 and 3, and exon 3, in all eight glioma samples and three established glioma cell lines tested [20]. The methylation patterns explained the down-regulation of SLC8A2 in gliomas, which could not be detected at any significant level. These findings suggest that methylation may play a key role in the transcriptional silencing of SLC8A2. SLC8A2 encodes a Na(+)/Ca(2+) exchanger, which contributes to intracellular Ca(2+) homeostasis, and its expression is restricted to normal brains. Our identification of SLC8A2 as a bivalent tumor suppressor gene supports the argument that SLC8A2 is a good target for therapy in glioma.

We found that another solute carrier gene, SLC17A7, is also a candidate bivalent tumor suppressor gene. As its role as a tumor suppressor has not been studied previously, we conducted further analysis. We found that SLC17A7 is down-regulated at both the protein and RNA levels in GBM compared with normal brain tissues (Figure 5), suggesting that it might be a tumor suppressor gene whose expression is down regulated in GBM to reduce its tumor suppressor activities. Indeed, overexpression of SLC17A7 in GBM cells reduced proliferation, migration and invasion potential of GBM cells (Figure 6), making SLC17A7 a potential target for GBM.

## MATERIALS AND METHODS

### Chromatin immunoprecipitation

Chromatin immunoprecipitation (ChIP) was performed using the SOLiD ChIP-Seq kit (ABI, Foster City, CA) according to the manufacturer's protocol. Antibodies against H3K4me3 (Millipore catalog no. CS200580), H3K27me3 (Millipore catalog no. 07–449), and H3 (Abcam catalog no. ab1791) or the control antibody normal Rabbit IgG (SOLiD ChIP-Seq Kit, ABI, Foster City, CA) were used for each IP as appropriate, and an additional IP was performed as a ‘no antibody’ control.

Purified ChIP DNA was validated by quantitative PCR using the 7900HT Sequence Detection System (ABI, Foster City, CA) with primers corresponding to Alu, GAPDH, SAT2, and ABCB1, using SYBR Green PCR master mix (ABI, Foster City, CA).

### ChIP-seq

ChIP DNA samples were used for SOLiD fragment library construction with the SOLiD Fragment Library Barcoding Kit Module 1–16 (ABI, Foster City, CA) and as described in the SOLiD Fragment Library Barcoding Kit protocol (Rev. B 04/2010). The Agencourt AMPure XP Kit (Beckman Coulter, Brea, CA) was used for purification steps after end repair, adaptor ligation, and PCR as described in the SOLiD ChIP-Seq Kit Guide (Rev. Date 18 March 2010). ChIP-Seq fragment libraries were analyzed on a Bioanalyzer using a High Sensitivity DNA Kit (Agilent, Santa Clara, CA) and quantified by qPCR using the SOLiD Library TaqMan Quantitation Kit (ABI, Foster City, CA). Following quantitative PCR quantitation, 8 ChIP-Seq fragment libraries (barcode1–8) were pooled for full-scale emulsion PCR with a 2.0 pM final library concentration, followed by enrichment of templated beads and 3′-end modification as described in the Applied Biosystems SOLiD 3 Plus System Templated Bead Preparation Guide (October 2009).

Beads were quantitated using the SOLiD Bead Concentration Chart and spectrophotometry following ePCR and 3′-end modification. Fifteen million beads were used for a WFA run to validate the prepared beads as described in the Applied Biosystems SOLiD 3 Plus System Instrument Operation Guide (October 2009).

Using the WFA result as a reference, 100~110 M beads were transferred to each well of the 4-well chamber as necessary, with 2 wells being used for 1 pooled library (barcode 1–8). All of the steps for bead deposition and instrument preparation for the ChIP-Seq run were performed as described in the Applied Biosystems SOLiD 3 Plus System Instrument Operation Guide (October 2009).

### ChIP-seq data analysis

The sequence tags were aligned to the human genome (hg19) using the Bowtie program, with the parameters -n 3 –best –strata -m 1 -p 4. Samtools was used for file format conversions. We then used the SICER program to identify H3K4me3- and H3K27me3-marked peaks. The parameters for identifying H3K4me3 peaks were as follows: window size 200, gap size 200, and FDR 1E3. The parameters for identifying H3K4me3 peaks were as follows: window size 200, gap size 600, and FDR 1E3. Overlapping peaks within 1 kb were combined using Bedtools. For annotation, the closest genes within 1 Mb of the peaks were used.

### Western blot analysis

20ug of cell lysates were prepared using IP Lysis Buffer (Pierce, 87787) plus protease inhibitor (Roche, 04693124001) and phosphatase inhibitor mixtures (Roche, 04906845001), proteins were separated by polyacrylamide gels (Novex, NP0323BOX) containing 0.1% SDS (SDS-PAGE), and transferred to PVDF membranes (Millipore, IPVH00010). Membranes were blocked overnight in 5% milk at 4°C. Immunoblots were probed for 2 hr at room temperature in 5% milk/TBST with the following primary antibodies: Anti-VGluT1 antibody (Abcam, ab77822) (VGluT1 is an alias of SLC17A7) at 1ug/ml, and anti-GAPDH (Millipore, MAB374) at 1ug/ml. Immunoblots were washed 3x for 10′ in TBST. Anti-rabbit (Abcam, ab150078) and anti-mouse (Abcam, ab150105) were used for 1 hr at room temperature and diluted 1:2000 in 5% milk/TBST. Immunoblots were washed 3x for 10′ in TBST, dried at RT, and visualized using a LI-COR Odyssey.

### Cell proliferation assays

T98 and U87 cells from ATCC (Manassas, VA) were plated at the density of 1 × 10^6^ cells in 100mm dish overnight. An adenovirus expression construct containing the SLC17A7 gene (Ad-hSLC17A7) was constructed and the adenovirus packaged by the Vector Biosystems, PA. Cells were infected with the adenovirus Ad-hSLC17A7 at 2 MOI for 24 hrs. Adenovirus containing empty cloning vector was used as a negative control. Cells were detached and plated into 96 well plates at 5 × 10–3 cells/well and incubated at 37C till assayed for cell proliferation with Toxcount (Activ Motif, CA) over 96 hours. Counting of live cells tagged green fluorescence was measured by Isocyte Laser Scanning Cytometer (Molecular Devices, CA).

### Cell migration assays

Cells infected with adenovirus for 24 hrs were detached and washed with serum-free media. 2 × 10^5^ cells were resuspended in serum-free media and loaded into 8um membrane inserts (Corning, MA) and placed over media containing 10% fetal bovine serum. The cells migrated to the lower compartment over the time points of 24, 48, 72, and 96 hrs were scored by Toxcount (Active Motif, CA).

### Cell invasion assays

For cell invasion assays, Matrigel (BD, CA) coating of 8um membrane inserts were used according to the manufacturer's instruction. In brief, cells that were infected with adenovirus for 24 hrs were detached and washed with serum-free media. 2 × 10^5^ cells were resuspended in serum-free media and loaded into 8 um membrane inserts coated with Matrigel (Corning, MA) and placed over media containing 10% fetal bovine serum. The cells migrated to the lower compartment over the time points of 24, 48, 72, and 96 hrs were scored by Toxcount (Active Motif, CA).

## CONCLUSION

In summary, being the first of its kind, our data of the global patterns H3K4me3 and H3K27me3 in glioma stem cells and astrocytes, and the differential expression patterns between the two, would be a useful resource for the research community. We further identified 317 bivalent genes that are enriched in WNT signaling and HOX gene family and in biological processes (tissue development and organ development) and cell differentiation, suggesting that bivalent genes might be important for glioma stell cell biology. Finally, we showed that SLC17A7 and SLC8A2 are two bivalent tumor suppressor genes whose expression were down-regulated in GBM tissues compared with normal brain tissues. Functional analysis showed that overexpression of SLC17A7 in GBM cells reduced proliferation, migration and invasion potential of GBM cells, suggesting SLC17A7 is a potential target for glioma therapy and warrants further investigation.

## SUPPLEMENTARY TABLES
















