# Supplementary material for: Global analysis of H3K4me3 and H3K27me3 profiles in glioblastoma stem cells and identification of SLC17A7 as a bivalent tumor suppressor gene
Source: Oncotarget. 2015 Jan 22;6(7):5369–81. doi: 10.18632/oncotarget.3030 (PMC4467155; doi:10.18632/oncotarget.3030)
Supplement: Supplementary file 1 [file oncotarget-06-5369-s001.pdf]

SUPPLEMENTARY TABLES

Supplementary Table 1: Number of reads sequenced

	H3k4me3	H3K27me3	H3	input
astrocyte	2865479	13355775	9098525	7974469
G179	6906513	7775976	5427855	6030860
SN143	5119858	7971838	6354351	5818599
SN175	3229614	8743216	10905116	12037890
SN179	5630050	8950739	4886216	6651960
SN186	4439975	7723917	4756285	4814305
SN187	5172552	9693451	8677823	8652463
SN201	3528937	7873323	7607604	6170177
SN207	8712172	7752806	8885977	7749984

Supplementary Table 2: Genes with H3K4me3 marks in more than 4 of 8 GSCs

Supplementary Table 3: Genes with H3K27me3 marks in more than 4 of 8 GSCs

Supplementary Table 4: Comparisons of H3K4me3 and H3K27me3 profiles between GSCs and human ESC (hESC)

Supplementary Table 5: H3K4me3 peaks identified in astrocyte cells and the comparison with those found in GSCs

Supplementary Table 6: H3K4me3 peaks identified in astrocyte cells and the comparison with those found in GSCs

Supplementary Table 7: Bivalent genes found in more than 4 of 8 GSCs

Supplementary Table 8: Bivalent genes for astrocytes
